# Mechanics and Dynamics of Intractable Constipation

**Published:** 1909-06-26

**Authors:** 


### Surgery.

### MECHANICS AND DYNAMICS OF INTRACTABLE CONSTIPATION.

### A REVIEW OF MODERN SURGICAL MEASURES.

To elucidate the peculiarly untroubled temperament of one of the women in his latest novel, Mr. H. G. Wells remarks that " she was—indeed she was magnificently—eupeptic." The range of the distinguished author's knowledge includes a sufficiently intimate acquaintance with physiology for us to be sure he meant by this phrase something more than mere perfection of the chemical processes of digestion, something more than uniformly good absorption and assimilation; he had in mind also complete freedom from the curse of modern civilisation, irregular and inadequate or delayed evacuation of waste products. Indeed, it might be postulated that an efficient daily action of the bowels was the first requisite of this euphonic " eupepsia."

Mr. Arbuthnot Lane, in his brochure on the " Operative Treatment of Chronic Constipation,"* says mildly that " indigestion in one form or another may be regarded as a usual concomitant of delayed and obstructed drainage." Of course Mr. Lane is driving at a condition of things far removed from what is ordinarily understood by chronic constipation. He is thinking of the exceptional cases in which there is extreme alteration of the mechanical conditions normal to the large intestines; of a dilated cæcum bulging down to the floor of the pelvis only suspended by a kinked and adherent appendix, or by " acquired mesenteries "; of an ascending colon unduly bound down to the outer and back wall of the abdominal cavity; of flexures rigidly suspended at a high level; of transverse colon descending in V-shape to rest on the top of the cæcum; of sigmoid flexure distorted and constrained by those bands of peritoneum that undoubtedly are occasionally to be found uniting the adjacent limbs of loops of bowel closely bent upon themselves.

It is very fairly well established now, that mere short-circuiting of the ileum into the upper end of the rectum, whether by lateral anastomosis or by implantation after transverse division, cannot be regarded as a complete operation. The cul-de-sac of colon sooner or later gives serious trouble, whether by antiperistalsis from the sigmoid or by the accumulation and inspissation of its secretions, as Mr. Lane would have us believe. Drainage of the excluded portion does not suffice to secure atrophy, so there is nothing for it, if operation is deemed necessary, but excision of the gut. The magnitude of the operation is not the only danger; but desperate conditions demand desperate measures.

The utility of another much-discussed procedure is not yet fully proven. Appendicostomy, which is certainly not always the simple business described by its advocates, when it can be done, affords not only a means of washing through the colon and administering salts directly into the large gut, but also serves in some degree to anchor the cæcum up out of the pelvis—a most important point.

Mr. Lane has disarmed criticism of his ideas as to the ætiology of the bands, mesenteries, and adhesions he describes, by saying that we need not believe him unless we like. When he talks about the " materialisation in peritoneum of lines of force," even on the analogy of Wolff's law, there is some strain on the imagination; but when he talks about the causation of cystic disease of the breast and of the ovary by intestinal auto-intoxication, and predisposition to gastric ulcer and tuberculosis of the joints, hinting that prevention is the ideal to aim at, then we think his remedy, excision of the colon, might very well prove worse than the disease; and this in spite of the endeavours of Mr. Hey Groves and Dr. Cohendy to prove that the lower end of the sigmoid and the rectum can efficiently perform all the physiological functions of the whole large intestine. Such a condition of affairs, while it would not necessarily interfere with the hypothetical syphon action, the hydrostatics, as it were, of the large intestine, does upset the dynamics of colon function.

It is not quite clear how much of the disturbance is due to mere displacement and how much to constricting bands, for Hertz has shown by his Bismuth-*x*-ray investigations that in normal individuals in the upright position there is V-shaped dependence of the transverse colon, and that sharp kinking of the hepatic and splenic flexures, as seen in cases of visceroptosis, does not lead to delay of the contents at those points. Hertz found too that a considerable proportion of cases of constipation were really instances of deficient action of the rectum—" dyschezia," as he calls it. The contents of the bowel pass at the normal rate all the way to the sigmoid, and only suffer delay in the rectum. The presence of fæces in the rectum should excite the desire to go to stool, and it is probably continued neglect of this normal stimulus that often leads to inertness or passivity of the rectal musculature.

No aperients will do this class of case any good; enemata are the rational treatment. In a great many cases where there is real stagnation in the cæcum and ascending colon, thorough systematic treatment by massage and movements, exercises and enemata will do a great deal towards restoring function; but much time and patience are imperative, and cure in the sense of impunity from further attention is not to be expected.

Every now and then, however, cases are seen in practice wherein every effort is exhausted without success, and the patient is reduced to the terrible condition of physical and mental misery for which Mr. Lane finds salvation in excision of the colon. Lethargy, despondency, and irritability, mental and moral hebetude are the psychical expression of progressive auto-intoxication; the physical expression is found in steady loss of flesh, degeneration of the tissues, attacks of faintness, giddiness, neuralgia, or headache, and premature ageing.

* " The Operative Treatment of Chronic Constipation," by W. Arbuthnot Lane. Nisbet and Co., London. Quarto. Pp. 70. Illustrated.

